# Advances and Prospects in Stem Cells for Cartilage Regeneration

**DOI:** 10.1155/2017/4130607

**Published:** 2017-01-26

**Authors:** Mingjie Wang, Zhiguo Yuan, Ning Ma, Chunxiang Hao, Weimin Guo, Gengyi Zou, Yu Zhang, Mingxue Chen, Shuang Gao, Jiang Peng, Aiyuan Wang, Yu Wang, Xiang Sui, Wenjing Xu, Shibi Lu, Shuyun Liu, Quanyi Guo

**Affiliations:** ^1^Institute of Orthopaedics, Chinese PLA General Hospital, Beijing Key Lab of Regenerative Medicine in Orthopaedics, Key Laboratory of Musculoskeletal Trauma & War Injuries, PLA, 28 Fuxing Road, Haidian District, Beijing 100853, China; ^2^Anesthesiology Department, Chinese PLA General Hospital, 28 Fuxing Road, Haidian District, Beijing 100853, China; ^3^Medical College, Nankai University, Tianjin, 300071, China; ^4^Center for Biomedical Material and Tissue Engineering, Academy for Advanced Interdisciplinary Studies, Peking University, Beijing 100871, China

## Abstract

The histological features of cartilage call attention to the fact that cartilage has a little capacity to repair itself owing to the lack of a blood supply, nerves, or lymphangion. Stem cells have emerged as a promising option in the field of cartilage tissue engineering and regenerative medicine and could lead to cartilage repair. Much research has examined cartilage regeneration utilizing stem cells. However, both the potential and the limitations of this procedure remain controversial. This review presents a summary of emerging trends with regard to using stem cells in cartilage tissue engineering and regenerative medicine. In particular, it focuses on the characterization of cartilage stem cells, the chondrogenic differentiation of stem cells, and the various strategies and approaches involving stem cells that have been used in cartilage repair and clinical studies. Based on the research into chondrocyte and stem cell technologies, this review discusses the damage and repair of cartilage and the clinical application of stem cells, with a view to increasing our systematic understanding of the application of stem cells in cartilage regeneration; additionally, several advanced strategies for cartilage repair are discussed.

## 1. Introduction

Cartilage defects, the most common disease of joints, can cause swelling, pain, and subsequent loss of joint function [1]. The capacity for cartilage self-repair is limited due to its unique structure, as it lacks blood supply, nerves, and lymphangion; cartilage absorbs supplements mainly from the synovial fluid. Therefore, traumatic articular cartilage injury and early osteoarthritis (OA) cause pain, accelerate arthrosis, and cause severe dysfunction. Meniscus injury results in pain to patients, limits their movement, and can accelerate the occurrence and development of OA. Intervertebral disc cartilage injury is one of the leading causes of chronic back pain [2]. Cartilage injury and subsequent tissue degeneration can cause long-term chronic diseases; moreover, such damage consumes large amounts of medical resources [3]. However, the field of regenerative medicine has shown promising developments in the repair of damaged cartilage.

Seed cells are the key components of regenerative medicine, which leads to healing. Autologous cartilage is the gold standard for cartilage seed cells in regenerative medicine [4]. Autologous chondrocyte implantation (ACI) has been applied widely with confirmed clinical effects in terms of repairing cartilage defects [5, 6]. As the donor source for autologous chondrocytes is limited, cells must be amplified in monolayers in vitro before implantation to meet the requirements of repair. However, the expansion of monolayers can cause rapid chondrocyte dedifferentiation, leading to loss of the original cell phenotype [7]. Compared with normal cartilage cells, dedifferentiated chondrocytes are more likely to generate fibrous cartilage instead of hyaline cartilage; the latter has better biomechanical properties and is more durable. However, autologous cartilage transplantation requires a second surgical operation and increases the risk of injury to healthy cartilage in the donor area. Chondrocytes maintain their phenotype when cultured in vivo with cytokines in three-dimensional (3D) cultures [8, 9]. However, the clinical application of autologous chondrocyte repair is limited.

Stem cells have the potential for self-renewal and differentiation into multiple cell lines. Stem cells can be divided into three main categories: embryonic stem cells (ESCs), induced pluripotent stem cells (iPSCs), and adult stem cells [10]. ESCs are derived from the inner cell mass of blastocyst-stage embryos [11]. iPSCs can be derived from somatic cells via genetic reprogramming [12]. Adult stem cells are isolated from various adult tissues [13]. ESCs and iPSCs are pluripotent cells that differentiate into cells of all three lineages: ectoderm, mesoderm, and endoderm [14]. Adult stem cells are subdivided into multipotent and unipotent stem cells; unipotent cells can differentiate only into one cell type, such as satellite stem cells or epidermal stem cells. Multipotent cells can differentiate into several cell types in one lineage; for example, mesenchymal stem cells (MSCs) can differentiate into osteoblasts, chondrocytes, and fat cells [13]. The capacity for self-renewal and the potential for multiple differentiation of stem cells, such as ESCs, iPSCs, and MSCs, have been studied widely in the field of tissue regeneration. Furthermore, studies involving MSCs have been fully applied in the clinical setting [15]. In this review, we focus on the cartilage injury mechanism and treatment strategies and studies of stem cells in the field of cartilage regeneration.

## 2. Characterization of Cartilage Stem Cells

Based on the continuous damage-repair theory, Dowthwaite et al. were the first to describe cartilage stem cells (CSCs) on the surface of articular cartilage [16]. They discovered that CSCs and fibronectin have a close interrelationship. Furthermore, they showed that CSCs have high colony-forming efficiency and can express Notch 1, which plays an important role in the early steps in notch signaling, inducing chondrogenesis [17]. CSCs also exist in patients with end-stage OA [18], and cells with chondrogenic potential can migrate rapidly into damaged cartilage to downregulate the expression of Runx-2, an osteogenic transcription factor, and enhance the expression of Sox-9, a chondrogenic transcription factor. By regulating Runx-2 and Sox-9 to inhibit osteogenesis in the damaged cartilage, CSCs can facilitate chondrogenesis to improve cartilage self-repair [19]. The matrix synthesis potential of CSCs can be increased without altering their migratory capacity. While cartilage cells usually exist in the surface of cartilage [16, 18], Yu et al. found in 2014 that CSCs also exist in the deep zone of cartilage [20]; one-third of the surface area contains more cartilage stem cells than two-thirds of the deep area. Different regions have distinct gene expression patterns and specific differentiation potential, and these features may be related to the unique properties of the superficial and deep zone stem cells, thereby participating in articular cartilage homeostasis. Zhou et al. showed that, compared with chondrocytes, cartilage stem cells can overexpress chemokines such as interleukin-8 (IL-8) and C-C motif ligand 2 (CCL-2). However, during pellet cultivation, the content of glycosaminoglycan (GAG) is lower than that in cartilage cells [21]. CSCs overexpress chemokines, which increases immune cells. Furthermore, they mediate inflammation during the processes of cartilage damage and repair. After chondrogenic induction, collagen type II and aggrecan can be detected (but not collagen type X), which differs from bone marrow stem cells (BMSCs) [22]. However, collagen type X is closely related to cartilage degeneration and aging [23]. Meanwhile, inducing BMSCs and CSCs into chondrocytes in vitro is more likely to lead to cell hypertrophy. Several studies have reported that CSCs have a better effect than synoviocytes in terms of cartilage induction in vitro [21]. These results suggest that CSCs might have a stronger potential than MSCs (BMSCs and synoviocytes) for cartilage induction.

In 2016, Jiang et al. further studied human cartilage-derived stem cells and their potential in the clinical application of cartilage tissue repair [24]. Using in vitro and in vivo experiments, they compared the chondrogenic ability of cartilage stem cells that had been cultured under different conditions. They found that, in the low-density, low-glucose 2-dimensional (2DLL) medium, cartilage stem cells can differentiate into cartilage spontaneously, without being induced, which supports potential for clinical applications. One of the in vivo studies included 15 patients undergoing cartilage repair surgery with cartilage progenitor cells, each of whom had a 6–13 cm^2^ area of damage. Recently, Huang et al. found stem cells in the meniscus [25]. They compared several characteristics of meniscus-derived stromal cells, autologous BMSCs, and fibrochondrocytes, including their morphology, proliferation, colony formation, immunocytochemistry, and multidifferentiation. Both meniscus-derived stromal cells and BMSCs have a marker related to stem cells. In addition, they can differentiate into osteocytes, adipocytes, and chondrocytes in vitro. Compared with BMSCs, however, more meniscus-derived stromal cells can differentiate into cartilage, which means that they are more effective at chondrogenesis. Sang et al. isolated nucleus pulposus stem cells (NPSCs) and annulus fibrosus stem cells (AFSCs) from intervertebral discs [26]. Both disk stem cells can form colonies and express stem cell markers during early cell passages, and each type of stem cell has different characteristics that reflect the tissue function that they represent.

There is a gap between the cell phenotype and the potential for regeneration between regular articular cartilage and induced cartilage formed by differentiated cartilage stem cells. This difference affects the ability to form hyaline cartilage of high quality. However, compared with most stem cells, cartilage stem cells have a superior potential for cartilage regeneration [27]. Studies of CSCs are still in the early stage, and further studies are needed to understand their role in cartilage regeneration. Autologous stem cells face similar problems to those of ACI, such as risk of injury to healthy cartilage, the requirement for a second operation, and a series of issues that present during cartilage defect repair. In overcoming problems of cellular immune rejection or cells with low immunogenicity, allogeneic cartilage stem cells present an attractive approach for cartilage defect repair [24].

## 3. Chondrogenic Differentiation of Stem Cells

Stem cells have the potential for multiple differentiation and self-replication, making them an ideal choice for use as seed cells in cartilage tissue engineering. An important step in the tissue engineering of cartilage is the induction of stem cells (including ESCs, iPSCs, and adult stem cells) into chondrocytes. Through tissue engineering, ESCs can be induced to form chondrocytes that repair cartilage damage [54]. Because undifferentiated ESCs have a high risk for tumorigenicity and teratoma, it is important to use stable and effective culture conditions to amplify ESCs and induce them to differentiate into a specific chondrogenic lineage [55]. Many strategies have been applied to induce ESC differentiation into chondrogenic lineage [56], including (1) embryoid body formation, a strategy that imitates the early stage of embryonic development as the ectoderm, mesoderm, and ectoderm; (2) differentiation into MSCs, a method that takes advantage of the immune exemption features and higher security of MSCs, which facilitates cartilage tissue engineering; and (3) the use of growth factors and cytokines such as members of the TGF-*β* family (e.g., TGF-*β*1 and TGF-*β*2), BMP family (e.g., BMP-2, BMP-4, and BMP-6), PDGF-bb, IGF-1, and sonic hedgehog protein (SHH). Several other strategies have been used that are similar to adult stem cell strategies, such as chondrocyte or fibrocyte coculture, 3D culture to change the cell microenvironment, hypoxia induction, and mechanical stimulation [54].

iPSCs can be derived from somatic cells through genetic reprogramming [57]. ESCs and iPSCs display self-replication and pluripotency, with iPSCs having distinct ethical advantages over ESCs. Originally, four factors—octamer-binding transcription factors 3 and 4 (Oct3/4), Kruppel-like factor 4 (Klf4), v-myc avian myelocytomatosis viral oncogene homolog (c-myc), and Sox-2—were identified in a mouse model as being involved in changing fibroblasts into iPSCs [57]. Of the four, Oct3/4 and Sox-2 are transcription factors, while Klf4 and c-myc are genes that are upregulated in tumors [10]. This discovery was a breakthrough in the stem cell field and provided a new tool in gene therapy and tissue engineering. Since then, somatic cells, fibroblasts, and chondrocytes have been reprogrammed successfully to become iPSCs and differentiate into chondrogenic lineage [58]. iPSCs derived from fibroblasts of skin can be induced into chondrocytes. Additionally, based on the HLA phenotype, it is possible to build an iPSC library that can provide allogeneic iPSCs. Cells from the library can be induced into chondrocytes to regenerate cartilage. This strategy is advantageous because it limits costs while offering wide coverage [59]. Compared with other iPSC lines, the iPSC line derived from chondrocytes can express higher quantities of aggrecan gene products [60]. In addition, the expression of cartilage-related genes does not differ from that of chondrogenic markers. iPSC technology offers a new and safe way to repair cartilage. This process will require optimization of the production process, a better understanding of the biological characteristics, and establishment of a differentiation strategy to achieve a productive and functional chondrocyte-like cell line.

MSCs are considered to be the most promising cells for cartilage regeneration by cell transplantation, and they have been applied clinically [61]. MSCs that differentiate into chondrocytes are induced by molecules, cytokines (which are mainly growth factors), and the microenvironment in cultured cells. Chondrogenesis from MSCs can be divided into three stages [62]. First, the stem cells condense and cell-to-cell interactions occur. MSCs begin to express adhesion molecules, such as N-cadherin, tenascin-C, and neural cell adhesion molecule (N-CAM). The condensation of MSCs is crucial during the early stage of chondrogenesis. Then, transcription mediators are activated, such as bone morphogenetic proteins (BMPs), Sox-9, PTHrP/IHH, and the FGF signaling pathways [63]. Finally, extracellular matrix (ECM) and precartilage cells are formed. Following the formation of precartilage, the perichondrial cells proliferate rapidly, secrete more ECM, and differentiate fully.

Mature chondrocytes localize at cartilage tissue. The ability of chondrocytes to maintain their phenotype is closely related to the conditions of their local microenvironment [64], including the type of 3D extracellular matrix, hypoxic conditions, mechanical loading, and specialized morphological structure [65]. Similarly, MSCs require specific conditions to differentiate into chondrocytes. The coculture of chondrocytes and MSCs is a new way to culture cells so that chondrocytes can induce MSCs, and MSCs can promote chondrocyte proliferation [66].

## 4. Cartilage Injury Mechanisms and Treatment

### 4.1. Articular Cartilage

Articular cartilage damage can occur through violent injury, chronic inflammatory disease such as rheumatoid arthritis (RA), or degenerative joint diseases such as OA. Several important mechanisms related to the occurrence and development of cartilage damage and degeneration include inflammation reactions that change the chondrocyte phenotype, the loss of ECM components, and damage and refactoring of the cartilage-bone unit [67]. Inflammatory cytokines play an important role in the progression of cartilage degeneration, and blocking some inflammatory cytokines can delay cartilage degeneration. Inflammatory cytokines are secreted by mononuclear cells, which induce hyperplasia of the synovial membrane [68]. Studies suggested that inflammatory reactions exist only in the synovial tissue, but recent studies have also confirmed the occurrence of cartilage inflammation. Chondrocytes are separated from the degenerated articular cartilage hypertrophy in vitro [69]. The change in the phenotype of chondrocytes prevents them from producing cartilage ECM components—such as proteoglycan and collagen type II, which are required to maintain the biological characteristics of cartilage cells. Conversely, chondrocytes can reduce the proportion of proteoglycan and produce more collagen type X, which is related to cell senescence [70]. Articular cartilage and subchondral bone form an inseparable organic cartilage-bone unit; in fact, damage and degeneration of articular cartilage are certain to cause subchondral bone destruction [71]. Moreover, the separation of articular cartilage and subchondral bone causes osteochondritis dissecans (OCD).

Treatment strategies for articular cartilage injuries include palliative treatment strategies, arthroscopic debridement and arthroplasty treatment strategies, and regenerative treatment strategies.

Palliative treatment strategies mainly include physiotherapy (thermal and electrical stimulation, high-intensity ultrasound, pulsed electromagnetic fields, millimeter waves, ultrasound, and low-level laser therapy), weight loss and muscle strengthening programs, and medications (glucosamine and chondroitin are used as treatments for cartilage defects, and although neither drug is used to alleviate the symptoms, they have been proven to reverse or suspend the progression of cartilage degeneration). Injection treatment strategies, compared with surgery, offer convenience and low risk. The injected material can have a direct effect on articular cartilage and remain in the articular cavity for a long time. Due to these characteristics, many different studies on articular cavity injection treatment strategies have been reported, pertaining, for example, to platelet-rich plasma (PRP) [72, 73], drug delivery strategies [74], polyphenol stabilization of cartilage collagen against degradation, action of the IL-1 receptor as an antagonist against lubricin metabolism and cartilage degeneration, the activities of rapamycin [75], alendronate [76], hyaluronic acid [77], bone morphogenetic protein-7 [78], and lidocaine [79], which reduce live chondrocytes and change the gene expression of COL II and aggrecan, and intra-articular steroid injections [80]. Arthroscopic debridement is used mainly in the middle-late stage of articular cartilage degeneration. Although arthroscopic debridement as a treatment of knee OA has been widely adopted as a surgical option, its efficacy has been controversial [81–83]. Arthroscopic debridement includes articular cavity flushing, meniscus partial nephrectomy, the removal of loose bodies, removal of the synovial membrane, chondroplasty, and osteophyte resection. Studies have shown that arthroscopic debridement can relieve short-term symptoms, especially in patients with OA with acute pain and patients with loose bodies in the articular cavity. Arthroplasty has been used widely in the treatment of late-stage articular cartilage lesions, with replacement usually being of the knee or hip [84].

### 4.2. Meniscus

The meniscus is composed of lateral fiber and medial transparent chondroid tissues. It disperses the pressure between the tibia platforms and the femoral condyle. Damage to the meniscus is often due to direct violence and can also reflect chronic degeneration [85]. Like cartilage injury, meniscus injury shows limitations in self-repair. Only the lateral fiber, which has a blood supply, can be stitched, but damage to this fiber is quite rare. Apart from causing restricted movement of the knee joint, meniscus injury also changes the mechanical structure of the joint, accelerating cartilage degeneration therein. The most commonly used treatment for meniscus injury is arthroscopic suture or resection. This procedure can provide the best mechanical stability in the meniscus and the strongest binding force in the damaged area. Meniscus injuries that are unable to be sutured are generally treated by meniscus merotomy and meniscus resection [86]. Allograft meniscus transplantation and synthetic materials have been applied clinically and have shown better prevention of knee joint degeneration compared with meniscus resection [87, 88]. Numerous reports describing the use of stem cell-associated tissue engineering to treat meniscal injury have demonstrated advantages in meniscus regeneration, showing promise for future meniscus injury treatments [89, 90].

### 4.3. Intervertebral Disc

Many patients experience back pain (lifetime prevalence of up to 84%) [91]. Although back pain is a complex disease that can be affected by multiple factors, the majority of back pain in patients is caused by acute injury and degeneration of the intervertebral disc [92]. The intervertebral disc is formed by the inner core of the nucleus pulposus (NP) and the annulus fibrosis, which surrounds the NP. The former consists of chondrocyte-like intervertebral disc cells, unarranged collagen, and gel-like matrix components that are rich in proteoglycans. NP consists of parallel collagen fibers that form a circular arrangement and fibroblast-like cells [93]. Most acute injury due to mechanical force causes the annulus fibrosis to fall apart, and herniated NP oppresses the surrounding tissues, resulting in clinical symptoms. The pathogenesis of intervertebral disc degeneration is unclear; however, the increased rate of intervertebral disc cell death, loss of the ECM, change of phenotype of the intervertebral disc cells, and excessive inflammatory reaction are thought to play a key role in intervertebral disc degeneration [94].

Acute damage and degeneration of the lumbar joints are treated mainly by conservative or surgical treatments. If conservative treatment fails, surgery can be attempted to relieve the neurothlipsis. However, these interventions are focused on alleviating symptoms, rather than constituting a regenerative treatment. In recent years, the introduction and development of bioregenerative therapies have delayed intervertebral disc degeneration and allowed for tissue repair (i.e., ECM repair and regeneration). Bioregenerative therapies include gene therapy, targeting of biological factors, microRNA (miRNA) treatment [95], and tissue engineering based on stem cells [2, 61]. Among those bioregenerative therapies, the percutaneous injection of MSCs has been used clinically and has had a remarkable effect on improving discogenic pain [96]. These technologies can change the metabolism in the microenvironment of intervertebral discs and allow for intervertebral disc tissue regeneration, while maintaining the original biomechanics of the spine [97]. Although few clinical studies have examined MSCs injection, they have proved their safety and feasibility for improving discogenic pain. However, more clinical research is needed to support these benefits [2].

## 5. Regenerative Medicine in Cartilage Repair

### 5.1. Microfracture

The theory of microfracture in articular cartilage regeneration is based on the assumption that pluripotent stem cells, which are mainly BMSCs from bone marrow, can reach the damaged area by microfracture gap [98]. At the end of the procedure, it is important to assess whether there are fat granules overflowing from the bone marrow to verify the correct hole depth. Microfracture technology is reported to work best when the damaged area is 2–4 cm^2^ [99]. This technology exploits the multipotent capability of stem cells and accomplishes cartilage repair at low cost and with little surgical damage. However, the method causes fibrous cartilage formation in the repaired tissue, rather than the hyaline cartilage found in normal articular cartilage, which affects the biological performance [4, 100].

### 5.2. Mosaicplasty

Mosaicplasty, also known as autologous osteochondral transplantation, employs osteochondral plugs removed from a non-weight-bearing region of the joint to fill the damaged area. First applied in 1997, mosaicplasty is not strictly considered as a regenerative technology, and it also runs the risk of early failure of transplantation. Moreover, this technology can only repair damaged areas < 4 cm^2^ [101]. Cartilage that forms in the damaged area by autologous osteochondral transplantation is the same hyaline cartilage as normal cartilage. Mosaicplasty technology gives better results than microfracture repairs, but ACI in turn has more advantages than mosaicplasty [102].

### 5.3. Scaffold

The use of scaffolding can provide a 3D microenvironment for cartilage cells, solving the problem of chondrocyte differentiation in monolayer cultures. The scaffold prevents loss of chondrocytes, which grow in oriented scaffolding that simulates the normal arrangement of chondrocytes and thus forms a bionic structure [103]. By means of their mechanical properties, scaffolds can provide benefits for patients in early rehabilitation. Scaffolding is one of the most important components of tissue engineering [104]. Combined with various cartilage-related cytokines, it can be used to raise autologous stem cells to complete tissue repair status in the damaged region, including stem cells from blood, synovial fluid, synovial tissues, and cartilage. Stem cells loaded on the scaffold can be induced in vivo under a specific microenvironment. With the continuous development of material science and the application of 3D printing technology to the field of tissue engineering, cartilage repair combined with scaffold materials offers a promising future direction for articular cartilage, meniscus, and intervertebral disc repair [105, 106].

### 5.4. ACI and MACI

First applied in 1994, ACI has been reported widely with its satisfactory long-term, mid-term clinical results and magnetic resonance imaging (MRI) result [5]. Patients receiving ACI are generally <50 years old, and the area of damage is >1 cm^2^, and cartilage injury is a type caused by acute trauma [107]. Compared with preliminary stage, ACI has explored much more indications than before. There is quite a challenge that cartilage damage repair has been reported with better clinical effectiveness, such as in patients with failed cartilage repair surgery [108], early stage OA [109], older age [110], complex patellofemoral lesions [111], deep osteochondral lesions, and OCD [112]. Peterson et al. summarized 224 cartilage damage patients who had been treated by ACI in the past 20 years [113]. The subjective scores have a significant increase compared with preoperation time. The report also points out that 74% of the patients feel better or stable and 92% of the patients are satisfied with their treatment. Despite subchondral cysts, osteophytes, bone marrow edema, and other common side effects, ACI still has an excellent clinical result in the long run. However, this procedure also has several shortcomings, such as a second incision during gaining periosteal patch, hypertrophy in the repair area, and chondrocyte leakage [114]. It has been reported that utilizing collagen I or III membrane instead of periosteal patch can avoid a second incision and reduce the incidence rate of hypertrophy. MACI can avoid the cell leakage problem with the 3D culture of the cell. But no matter ACI or MACI, the chondrocyte phenotype maintenance is still a formidable issue during cell culture. Compared with prolonged monolayer culture in ACI, MACI can provide a 3D-culture microenvironment for chondrocyte adhesion, proliferation, and matrix secretion to maintain the chondrocyte phenotype [115]. It has been reported that 3D-culture microenvironment [65] and coculture [116] of stem cells with chondrocytes can do better in chondrocyte phenotype maintenance, which is the key point to determine the clinical effects of ACI and MACI, which needs more studies in the future.

### 5.5. Stem Cells and the Effect of Stem Cells on Cartilage Repair

In the past decade, stem cell-based treatment has been applied widely, and the number of studies on this topic has increased rapidly. Today, such treatment is an important branch of regenerative medicine. Stem cells have two effects: they have the potential for multiple differentiation and they have paracrine and immunomodulatory abilities, which are both important features in cartilage regeneration using MSCs [117, 118]. The fact that stem cells can differentiate into cartilage cells and that a scaffold can be utilized for cell attachment makes this system amenable to cartilage tissue engineering with stem cells in the clinic. Laboratory studies and clinical evidence show that stem cells are an efficient method for treating traumatic bone-cartilage injury [119]. Although the application of stem cells combined with scaffold materials, by using tissue engineering technology, can achieve a satisfactory repair effect, no studies have shown that the repair effect of stem cells is better than that of chondrocytes. The application of stem cells combined with scaffold, for tissue engineering of traumatic cartilage damage, has a satisfactory effect, but little success has been reported in terms of the repair of OA cartilage degeneration.

This treatment is based on the paracrine and immunomodulatory effects of stem cells. Most stem cell OA treatments involve injections to insert stem cells into the damaged area of the articular cavity. Meniscus injury is treated with articular cavity injection [120, 121], while intervertebral disc damage is treated with local injection [122, 123]. Although the mechanism is not fully understood, the effect is clear, especially for the treatment of OA. Many pathological reports and randomized controlled trials have demonstrated therapeutic effects. Stem cells secrete mediators that promote endogenous growth, stimulate self-proliferation of progenitor cells, and inhibit chondrocyte apoptosis or cartilage degeneration, achieving cartilage regeneration and cartilage protection [124]. In addition, several studies have shown that the inflammatory response in the injured area inhibits damage repair by endogenous stem cells or progenitor cells (such as cartilage stem cells) [125].

## 6. Clinical Applications of Stem Cell Therapy in Cartilage Repair

Compared with ESCs and iPSCs, adult stem cells are more secure and are therefore applied first in clinical therapy. MSCs are the most representative adult stem cells and are used widely in clinical cartilage regeneration. MSCs can be derived from various sources, such as bone marrow, fat, placenta, umbilical cord blood, synovial membrane, peripheral blood, tendons, and cartilage. BMSCs, ADSCs, synovial mesenchymal stem cells (SMSCs), peripheral blood-derived mesenchymal stem cells (PBMSCs), and other stem cells have been applied in clinical cartilage damage repair with satisfactory results (Table 1).  Table 2 summarizes the results of a PubMed database search for clinical trials involving stem cells in cartilage regeneration, published from 2000 until the end of June 2016. Several recent studies have investigated allogeneic BMSCs for treating OA, demonstrating their safety and effectiveness in cartilage repair. In addition, ADSCs have been studied in recent years in terms of cartilage repair. Compared with BMSCs, ADSCs have certain advantages in the treatment of cartilage damage. Osteoporosis causes a decline in the quantity and quality of BMSCs, but ADSCs can be used to address this condition. The safety of cartilage damage repair is higher when the stroma vascular fraction (SVF) is not cultured in vitro. After liposuction surgery, adipose tissue, in the form of medical waste, can be reused. The most attractive reason for using PBMSCs is that they are easily acquired and require only one-step surgery for cartilage repair. Few studies have described the use of SMSCs and chondrocyte-derived progenitor cells (CDPCs) to repair cartilage damage, and further clinical tests are required to clarify their advantages and disadvantages. CDPCs originate from cartilage tissue and have a superior ability to differentiate into cartilage. Tissues requiring repair generally include the meniscus of the knee joint and talus cartilage; damage to these regions is limited mainly to cartilage damage or early OA. Cells can be delivered using a variety of methods such as simple direct injection of MSCs, or MSCs mixed with hyaluronic acid (HA), PRP, or glue, as well as MSCs combined with scaffold.

Despite years of research, the use of stem cells in cartilage regeneration has not met expectations. MSCs possess an intrinsic differentiation program for endochondral bone formation [126]. Although researchers seek to avoid the hypertrophic fate of MSCs, they cannot yet create articular hyaline cartilage without the hypertrophic chondrocyte phenotype [69]. This challenge must be overcome to enable better cartilage regeneration using MSC-based tissue engineering. In addition, the use of stem cells in cartilage regeneration is limited to untreated or multiplication cultured stem cells. Although the feasibility of using stem cells in cartilage regeneration has been proved, few clinical studies have been reported because the induced cells are unstable [127] (i.e., they degenerate readily and lead to tumorigenesis). Therefore, more studies are needed to prove the safety of using stem cells to induce cartilage.

## 7. Conclusions

Stem cells research is an important fundamental research topic in cartilage regeneration. Although the role of stem cells in cartilage regeneration is certain, the mechanism underlying this process in cartilage repair is not yet clear. The full range of limitations and possibilities, with respect to clinical application of various stem cells, remains to be established, but the advantages of stem cells seem obvious. MSCs are the most widely applied stem cells in the field of cartilage regeneration, and their safety and effectiveness have been demonstrated in basic research and clinical studies. There are many clinical examples of stem cells showing a satisfactory curative effect in cartilage damage repair, but larger sample sizes and longer follow-up periods in clinical studies are required to test the effectiveness and safety of stem cells for cartilage repair.

## Figures and Tables

**Table 1:** Recent clinical trials involving stem cells in cartilage regeneration.

Cell type	Cell source	Location	Injury type	Cell carrier	Cases (*n*)	Follow-up	Description	Results	References
CDPCs	Autologous, cartilage-derived	Knee AC	Cartilage defects	Collagen type I/III scaffold	15	12 months	Compared with BMSCs, the chondrogenic potential was better	Ectopic calcification and vascularization were not found in tissue biopsies of four patients. The clinical scores of all patients showed improvement; function improved and pain was relieved.	2016 [24]

BMSCs	Autologous	Knee AC	OA	Injection	3	5 years	Update of a previous study	Long-term follow-up of stem cell injection showed good prognosis for patients with early-stage OA.	2016 [28]

BMSCs	Allogenic	Knee AC	OA	Injection	BMSCs: 15 HA: 15	12 months	RCT	Compared to the HA group, the function recovery and quality of regenerated cartilage are meaningfully enhanced in the BMSC group.	2015 [29]

BMSCs	Allogenic	Knee AC and meniscus	OA	Injection	Low-dose: 18 High-dose: 18 HA: 19	2 years	Partial medial meniscectomy RCT	Knee joint pain was relieved, and MRI showed meniscus regeneration in the stem cell group.	2014 [30]

BMSCs	Autologous	Knee AC	OA	Injection	12	2 years	Update of a previous study	Pain was relieved after 1 year of treatment, which continued through year 2. MRI showed better quality of cartilage in year 2 compared to year 1.	2014 [31]

BMSCs	Autologous	Knee AC	OA Cartilage defects	Injection	HA + BMSCs: 28 HA: 28	2 years	RCT high tibial osteotomy + microfracture	Effectively improving both short-term clinical and cartilage repair tissue scores.	2013 [32]

BMSCs	Autologous	Ankle	Chondral defects	Collagen membrane	25	2 years	Matrix-associated stem cell transplantation	Good clinical scores and no complications.	2013 [33]

BMSCs	Autologous	Knee AC	Cartilage defects	Injection periosteal patch	Microfracture + BMSCs + HA: 35 BMSCs + patch: 35	2 years		Microfracture + BMSCs + HA are comparable to BMSCs + patch, but minimally invasive.	2012 [34]

SMSCs	Autologous	Knee AC + meniscus	Cartilage defects	Arthroscopic transplantation	10	37–80 months	10% autologous human serum used to expand cells	MRI scores, Lysholm score, and qualitative histology all show that SMSC transplantation is meaningful.	2015 [35]

ADSCs	Autologous	Knee AC	Cartilage defects	Arthroscopic	ADSCs + microfracture + fibrin glue: 40 Microfracture: 40	2 years	RCT	Radiologic and KOOS pain and symptom scores show a more meaningful improvement than that of the control group.	2016 [36]

ADSCs	Autologous	Knee AC	OA	Arthroscopic	ADSCs + fibrin glue: 20	2 years		Clinical and MRI scores show a significant improvement.	2016 [37]

ADSCs	Autologous	Knee AC	OA	Injection	SVF: 1,128	12–54 months		No serious side effects, infection, or cancer related to SVF.	2015 [38]

ADSCs	Autologous	Knee AC	OA	Injection	30	2 years	4.04 × 10^6^ stem cells	Effective for elderly patients with OA at the knee.	2015 [39]

ADSCs	Autologous	Knee AC	OA	Arthroscopic	ADSCs: 37 ADSCs + fibrin glue: 17	24–34 months		Arthroscopic and clinical outcomes were useful for OA in both groups. However, the ADSC + fibrin glue group had better ICRS scores.	2015 [40]

ADSCs	Autologous	Knee AC	Early OA	Arthroscopic	ADSCs + fibrin glue: 49	Mean 26.7 months		Patients > 60 years of age or having injury areas < 6 cm^2^ were not suitable for this treatment.	2015 [41]

ADSCs	Autologous	Meniscus	Meniscal tear	Injection	ADSCs + PRP + CaCl_2_ + HA: 1	18 months		Pain was alleviated. MRI at 3 months after treatment showed that the meniscal tear had almost disappeared.	2014 [42]

ADSCs	Autologous	Knee AC	OA	Arthroscopic	Knee: 37	24–34 months		The factors affecting the repair result were mostly large injury area and high BMI. The second arthroscopic view showed 76% nonregular repair.	2014 [43]

ADSCs	Autologous	Talus	Osteochondral lesions	Injection	Marrow stimulation: 26 SVF + marrow stimulation: 24	21.9 months		Marrow stimulation with SVF group showed better results than the marrow stimulation alone group.	2014 [44]

ADSCs	Autologous	Knee AC	OA	Injection	I: low-dose (3), medium-dose (3), high-dose (3) II: high-dose (9)	6 months	Low dose: 1 × 10^7^ Medium dose: 5 × 10^7^ High dose: 1 × 10^8^	No adverse events. The high-dose group showed better results than the other groups.	2014 [45]

ADSCs	Autologous	Knee AC	OA	Injection	ADSCs + PRP: 91	30 months		Safety of autologous SVF and percutaneous local injections was demonstrated by MRI and telephone follow-up.	2013 [46]

ADSCs	Autologous	Knee AC	OA	Injection	SVF + PRP: 18	24–26 months		ADSCs of the infrapatellar fat pad were useful for relieving articular pain and improving knee joint function.	2013 [47]

ADSCs	Autologous	Talus	Osteochondral lesions	Injection	Microfracture: 30 Microfracture + ADSCs: 35	21.8 months		Among patients above 50 years of age, the effect of marrow stimulation + ADSCs was better than marrow stimulation alone. >109 mm^2^ lesion size and existing subchondral cyst showed better regeneration results.	2013 [48]

ADSCs	Autologous	Knee AC	OA	Injection	ADSCs + PRP: 25	12 months	1.89 × 10^6^ ADSCs, 3 mL PRP	ADSCs of the infrapatellar fat pad were useful for releasing articular pain and improving knee joint function.	2012 [49]

PBSCs	Autologous	Knee AC	Chondral lesions	Open surgery	1	7.5 years	Periosteal flap + patellofemoral realignment	CT and MRI showed better results. Eight months after the surgery, the second arthroscopy showed that the new-growth cartilage had a smooth surface. The patient returned to practicing Taekwondo.	2014 [50]

PBSCs	Autologous	Knee AC	Early OA	Injection	5	6 months	PBSCs + HA + growth factor + microfracture	No adverse events and all clinical scores improved.	2013 [51]

PBSCs	Autologous	Knee AC	Chondral defects	Arthroscopic	Microfracture + HA: 25 PBSCs + microfracture + HA: 25	2 years	RCT	PBSC group has a better quality of newborn cartilage than the control group on histological and MRI assessments.	2013 [52]

PBSCs	Autologous	Knee AC	Chondral defects	Open surgery	52	6 years	Collagen membrane	PBSCs are an effective way to repair large cartilage lesions. This method can be used as an alternative to ACI.	2012 [53]

**Table 2:** Types of stem cells used clinically for cartilage regeneration past and present. This table shows the PubMed database search results for clinical trials involving stem cells in cartilage regeneration, published from 2000 until the end of June 2016 (number of papers).

Year	Cell type					Total
	BMSCs	ADSCs	PBSCs	SDSCs	CDPCs	
2002	1	0	0	0	0	1
2004	1	0	0	0	0	1
2005	1	0	0	0	0	1
2007	2	0	0	0	0	2
2008	1	0	0	0	0	1
2010	2	0	0	0	0	2
2011	2	1	1	0	0	4
2012	2	1	1	0	0	4
2013	2	2	2	0	0	6
2014	2	4	1	0	0	7
2015	1	4	0	1	0	6
2016	1	2	0	0	1	4

Total	18	14	5	1	1	39

## Abbreviations

AC: Articular cartilage
ACI: Autologous chondrocyte implantation
ADSCs: Adipose-derived stem cells
BMI: Body mass index
BMSCs: Bone mesenchymal stem cells
CT: Computed tomography
CDPCs: Chondrocyte-derived progenitor cells
HA: Hyaluronic acid
KOOS: Knee injury and osteoarthritis outcome score
ICRS: International Cartilage Repair Society
MRI: Magnetic resonance imaging
OA: Osteoarthritis
PBSCs: Peripheral blood stem cells
PRP: Platelet-rich plasma
RCT: Randomized controlled trial
SVF: Stroma vascular fraction.
